# On the Perceptive Power of the Spinal Cord, as Manifested by Experiments on Cold-blooded Animals

**Published:** 1858-05

**Authors:** George Paton

**Affiliations:** Galt, Canada West


ART. I.—*On the Perceptive Power of the Spinal Cord, as manifested by Experiments on Cold-blooded Animals.* By GEORGE PATON, M.D., of Galt, Canada West.

IT is considered a well-established fact in metaphysics, that the nerves are the medium through which we obtain a knowledge of the external world—that the impressions produced on their extremities are conveyed by them to the brain, the seat of the mind—and that an animal, by means of its senses, becomes acquainted with the various objects by which it is surrounded. And physiologists have considered it of much importance to ascertain if the power of recognizing impressions be connected with the spinal cord, or exclusively seated in the brain: in other words, what is the real state into which an animal is reduced after removal of its cerebral lobes? M. Flourens, in his experimental researches into the functions of the nervous system, has devoted much attention to this question, and endeavored to give it a precise and definite form. He carefully removed the cerebral lobes of an animal, and observed the phenomena. After the operation, the animal appeared to be thrown into a state of stupor, and no longer manifested spontaneous movements. In the language of Cuvier, "the animal so mutilated is quite drowsy, and has no will of its own; it makes no spontaneous motion, but when struck or irritated it rouses itself as from a sleep. In whatever position it is placed, it preserves its equilibrium. If laid upon its back, it rises; it walks if pushed. When it is a frog, it jumps if it is touched; when a bird, it flies if thrown up into the air; it struggles if annoyed; if water is thrown upon its back it swallows it." An animal in this condition will live several months, if properly fed and protected, but its food and drink always require to be placed upon its tongue that they may be swallowed.

M. Flourens considers that an animal in this state neither feels nor perceives the stimulus to which it is subjected; that it has lost all sense of touch; and that the movements depend on a principle of excitability possessed by the spinal cord. From which he concludes that the cerebrum alone is the seat of sensation and perception. And in maintaining these views, he considers that the optic lobes or corpora quadrigemina are the continuation and termination of the spinal cord and medulla oblongata.

Dr. Marshall Hall proceeded further than M. Flourens in accounting for the movements of an animal deprived of its cerebrum. Pursuing the views that had been advanced by Prochaska respecting the reflex action of the spinal cord—that an impression on the extremities of the *afferent nerve* is conveyed by that nerve to the medulla, and reflected by the *efferent* or *motor nerve* of the part; and that this is the principle on which the movement is effected; he reduced this class of movements into a system, and illustrated their importance in the functions of the body. He showed that the contraction of the iris, the act of deglutition, the associated movements of sneezing, coughing, defecation, &c., all depended on the principle of reflex action; that it was the impression on the *afferent* or *sensory nerve*, conveyed to the medulla and reflected by the *efferent* or motor nerves of the part, by which the movement was produced, each being excited by its appropriate stimulus, as contraction of the iris by the application of light to the eye, &c. But he generalized these views so far as to maintain that this is the character of all those movements that are witnessed in decapitated animals; and concluded that the spinal cord is a distinct system in itself, and the seat of reflex action; and that the brain is the centre of the senso-volitional movements; each organ performing its specific function in the animal economy.

M. Granger endeavored to corroborate the views of Dr. Hall, by his investigations into the structure of the spinal cord. He stated that he could trace some of the fibres of the anterior and posterior roots of the nerves into the cineritous matter of the cord, which he considered to be connected with the reflex function; and that the other nervous fibres did not enter the substance of the cord, but proceeded, as the medullary fibres, to the brain, and that these constituted the senso-volitional system. Hence, each spinal nerve contained both reflex and senso-volitional fibres.

1. In reference to these views, we may state that the theory of a true spinal system is decidedly opposed to the movements performed by cold-blooded animals after removal of the cerebrum. We shall afterwards see that these movements indicate a power resident in the spinal cord distinct from that of reflex action.

2. There are circumstances connected with the reflex movements of the body which do not coincide with the theory of Dr. Marshall Hall.

We admit that an important class of movements of the body depend on reflex action; that contraction of the iris, deglutition, respiration, &c., are produced by the impression on the *afferent nerve* being conveyed to the medulla, and reflected by the efferent or motor nerve of the part; and that these movements take place independently of sensation. But if by the reflex function it be understood that the motor nerve is always excited by the afferent nerve without the intervention of sensation, then we dissent from the view, for there is a class of reflex movements into which sensation appears to enter as an essential element, as necessary to constitute the act.

The associated movements of sneezing, coughing, the closure of the eyelids on the application of a strong light to the eye, &c., are of this character, the impression being conveyed by the sensory nerves of the cord to the medulla and reflected by the motor nerves. And this implies a connection with the sensory columns of the cord—a doctrine opposed to that of a true spinal system. Besides, the muscles of voluntary motion seem susceptible of reflex action, through the medium of sensation, as when the limbs start up involuntarily by being suddenly pricked with a pin or similar mode of irritation. Indeed, what appears to constitute a reflex movement, is that the impression, on being conveyed to the medulla, should be immediately reflected by the motor nerve independently of the will. It is the manner in which the motor nerve is excited, and not the mode in which the impression is conveyed, on which reflex action depends.

At the same time there can be no doubt that cases have occurred, where, from disease of, or pressure on, the spinal cord, the patient had lost all power of voluntary motion in his limbs, and reflex action could be excited in them by irritation of the integuments. Some of these cases have come within our own observation, and others have been related by different members of the profession, in which, after the loss of sensation and voluntary motion in the lower extremities, the limbs started up involuntarily and continued to be agitated for some time, by tickling the soles of the feet. And during this period the patient neither felt the irritation, nor the application of heat or cold to the integuments. But in all these cases it was only a part of the cord that was affected; and the movements are very different from the perceptive movements manifested by an animal after removal of the cerebrum.

The theory of an excito-motory system, brought forward in explanation of these facts, has been considered premature, and as by no means resting on data sufficient to command the general assent of the profession. It does not follow, as we shall afterwards see, that, because reflex movements may be excited in the lower portion of the cord in cases of disease, that this is the specific function of the whole of the cord, after ablation of the cerebrum. And the investigations into the structure of the spinal cord, which were formerly adduced in support of these opinions with as much boldness as if they had been mathematical truths, cannot be relied on, as they appear to have proceeded on previously assumed views, rather than to have been directed by strict observation and a rigid induction of facts. The recent microscopic observations of other physiologists respecting the structure of the spinal cord, differ materially from those propounded by M. Granger in support of Dr. Marshall Hall's theory.

Schrœder Van der Kolk, in his investigations into the minute structure and the functions of the spinal cord,* states "that several distinct vertical columns of multipolar ganglion cells exist in the cord, extending throughout its whole length; that they are most abundant in the anterior horns of the gray substance, then between the anterior and posterior horns, and lastly in the posterior horns themselves;" that they increase at the cervical and lumbar portions of the cord, where the relative plexuses of nerves are given off, and that a communication exists among the ganglion cells throughout the cord by fine nervous filaments.

He also states that the motor nerves take their origin from the ganglion cells of the cord; that the motor nerves spring from *the cord,* and especially out of the ganglion cells of the anterior horns; and that the anterior medullary fibres of the cord are the channels of communication between the brain and these ganglion cells, and by which the mandates of the will are conveyed to the motor nerves of the cord; "for the innermost longitudinal fibres can be seen to bend toward the gray substance and pass into the ganglion cells." And he considers that the sensory nerves of the cord give off one set of fibres which proceed directly to the ganglion cells, and that the other fibres ascend as posterior medullary fibres to the brain.

From this account of the minute structure of the spinal cord, it appears that *the whole of the motor fibres of the nerves* take their origin from the ganglion cells of the cord, and that no portion of them arises from the cerebrum, as was formerly supposed; that the anterior medullary fibres are the medium of communication between the brain and the ganglion cells, through which fibres the influence of the will is conveyed to the motor nerves; and that the reflex action is produced through the medium of the fibres of the posterior spinal nerves that pass directly to the ganglion cells from which the motor nerves arise; but that the sensory nerve-fibres do not enter the substance of the cord, but ascend as the posterior medullary columns, and convey impressions to the cerebrum as the seat of perception. But he considers it very probable that sensation has its proper centre in the medulla oblongata.

Dr. Lenhossek, in his researches into the central nervous system of man,† maintains that the primitive fibres of the root nerves, whether *motory* or *sensory,* simply traverse the white substance of the spinal cord, and that no portion of them bends itself upward to proceed to the brain, with the longitudinal fibres; but that they take their immediate origin from the

* Anatomical and Physiological Inquiry into the Minute Structure, and the Functions of the Spinal Cord. By J. L. C. Schrœder Van der Kolk. Amsterdam, 1854.

† New Researches into the Minute Structure of the Central Nervous System in Man. I. The medulla spinalis and bulbus rhachiticus. By Joseph V. Lenhossek, M.D., &c., Professor of Anatomy and Legal Medicine at the Imperial and Royal Lyceum in Klansenburgh. Vienna, 1855.

ganglion cells, the purely motor fibres springing from the anterior horns, and the purely sensory fibres from the posterior horns of the gray substance, and communicate by means of the middle layer of the ganglion groups, as these are seen to dispense filaments in both directions. And he also states that the primitive fibres of the medullary substance are more slender than those of the roots of the nerves, and appear to issue at very acute angles from the gray matter, and pursue a longitudinal and parallel course uninterruptedly to the brain.

Other microscopic observers maintain similar views, not merely in reference to the spinal nerves originating in the ganglion cells of the spinal cord, but that the fibres from the ganglion cells pass into the longitudinal fibres of the white substance, and proceed, as the medullary columns, to the brain.

These researches into the structure of the spinal cord must, in a great measure, supplant the views of Marshall Hall and Granger, as they show that all the roots of the motor nerves, including the senso-volitional fibres, take their origin from the gray substance of the spinal cord. And though a discrepancy of opinion exists respecting a portion of sensory fibres proceeding to the cerebrum, or terminating in the spinal cord, and it must be a matter of great difficulty, from the delicate structure of that organ, to determine by experiment the specific functions of particular fibres, yet one thing is certain: these researches have advanced us an important step in physiological science, in showing us that the spinal cord is intimately connected with the volitional movements of the body.

But it is not the minute structure of the spinal cord that we have to determine in order to establish its perceptive power, though that is of great importance in itself, and may be urged in corroboration of the doctrine. But it is the specific functions of the cord as manifested by an animal after ablation of the cerebrum, or, in other words, the movements which an animal in this state is capable of performing. And we maintain that an element enters into these movements perfectly distinct from that of reflex action, and which cannot be accounted for on any other principle than that the animal manifests distinct power, and exerts a definite control over the muscles of voluntary motion in taking cognizance of the irritation to which it is subjected.

And in determining the character of a reflex as distinct from that of a perceptive movement, it is to be observed, that a reflex movement being produced by the impression on the afferent nerve conveyed to the medulla, and reflected by the motor nerve, always takes place in the same manner—admits of no change or variation—and may be termed a uniform sequence of that which preceded it. Hence there can be no evidence of design, nor of any determinate movement exerted at the moment to the attainment of a specific object; the adaptation of means to ends connected with these functions of the body being inherent in the structure of the part, and not dependent on the will of the animal.

But in perceptive movements, when the impression on the afferent nerve reaches the medulla, the motor nerve is excited by a distinct act of volition, and the movements performed are varied in accordance with the circumstances of the case and the degree and mode of irritation; for the muscles on which these movements depend are not limited or restricted in their action, but subject to the control of the will for the very purpose of accomplishing our desires and intentions. Hence they are termed the muscles of voluntary motion—engaged in walking, leaping, the varied and combined movements of the limbs—of the larynx, &c.; and every specific act performed by this class of muscles, in which we witness design and adaptation of means to ends, must be considered the effect of a controlling power or agency, as there is nothing in the structure of the parts to account for it.

On the other hand, when reflex action is excited in the muscles of voluntary motion, as in the limbs by irritation of the integuments, the movements consist of a successive contraction and relaxation of the muscles of the part, similar to what we observe in convulsions or the application of galvanism; but there is nothing like design, or the attainment of a definite object in the movements.

From these statements, then, it will appear that in determining the character of the movements of an animal after ablation of the cerebrum, we must not limit our views to the existence or non-existence of sensation, which has too frequently been done by physiologists in discussing this question; for sensation, according to the strict and philosophical import of the term, expresses the mere feeling, or state in which a sentient being exists at the moment, without reference to the cause that produces it. And we maintain that we can have no evidence of sensation in an animal subjected to experiment, except so far as we observe proofs of perception or recognition of the stimulus. An animal may feel on being touched, but we can have no knowledge of the fact, unless it be manifested by the particular movements which it executes at the moment in response to the irritation, and this necessarily implies a certain degree of voluntary power exerted by the animal in regulating and controlling its movements to the attainment of an end—which phenomena we define by the term perceptive movements.

Another circumstance on which we must insist, of great importance in order to arrive at a proper decision on this subject, is, that we must base our views on strict observation and a rigid induction of facts. We cannot ascertain the functions of the brain or spinal cord, except so far as these are manifested by experiment or pathological research. And it is contrary to every principle of logic and sound philosophy to suppose that because an animal is deprived of its cerebrum it can give no evidence of perceptive power. We can assign no reason, so far as our investigations into the structure and functions of the nervous system are concerned, why the cerebrum should be possessed of this power, and not the medulla oblongata and spinal cord. The question is, What are the facts of the case? "The foundation of all human knowledge," says Mr. Stewart, "must be laid in the examination of particular objects and particular facts, and it is only so far as our general principles are resolvable into these primary elements, that they possess either truth or utility."

What, then, are the phenomena manifested by an animal after removal of the cerebrum, or brain? Have we evidence that it is capable of performing distinct perceptive movements?

*Experiment* 1.—I removed the cerebrum of a frog with great care, and observed the phenomena. Respiration continued. The frog no longer manifested spontaneous motion, but remained in a shallow vessel, amid a little water, with its hind legs drawn up, the posture that frogs assume when they rest; but on being irritated, moved with great vigor, and gave every indication of recognizing the stimulus. On being seized by the foot, it struggled much to be relieved, and on being freed, bounded from the grasp, and made several leaps before it became quiescent. On irritating the integuments of its left cervical region, it croaked; on repeating the irritation, it again croaked, and scratched the part with its left hind foot. On touching with the point of a needle the integuments over the right scapula, it raised up its right hind leg, and scratched the part with its foot. On continuing the irritation, it again raised up its right hind foot, and pushed away the instrument with force.

*Experiment* 2.—Removed the cerebrum of a frog with great care, and allowed it to remain quiescent till the effects of the operation had subsided. Respiration continued. The frog had lost the power of spontaneous motion, as it did not move from the place on which it was seated till irritated, but appeared most sensible to the touch, and gave every indication of recognizing a stimulus. When placed upon its back, it immediately turned upon its face, and made several leaps before it became quiescent. When I irritated slightly the integuments of its thorax on the right side, it pushed away the instrument with its right hind foot. On a stronger irritation being employed, it withdrew its body in the opposite direction, and leaped to a distance. When I compressed slightly one of the toes of its right fore foot, it withdrew its foot, and placed it below its abdomen. When I touched with the point of a needle the integuments of its left dorsal region, it scratched the part with its left hind foot. And when I touched with the point of a needle its right cervical region, it raised up its right hind leg and pushed away the instrument with its foot. In short, it comported itself as regards the sense of touch like an animal that had sustained no mutilation, the only difference being that a slight irritation was required to be employed before the perceptive movements were manifested.

*Experiment* 3.—I removed the cerebrum of a frog, dividing the medulla oblongata a little anterior to the origin of the par vagum. After the operation a few slight respirations were observed, which gradually ceased. I touched with the point of a needle the integuments of the right cervical region, and it quickly raised up its right hind leg and scratched the part with its foot. I irritated the integuments of its left dorsal region, and it raised up its left hind leg and pushed away the instrument with its foot, and then leaped to a distance, and made several successive leaps before it became quiescent. On irritating its right dorsal region, it withdrew its body in the opposite direction, and scratched the part with its right hind foot. In short, it gave every indication of recognizing the stimulus, and endeavoring to avoid it.

I have performed the same experiment on many other frogs with similar results. The animal, after ablation of the cerebrum, losing the power of spontaneous motion, but continuing most susceptible to every irritation, and performing distinct perceptive movements in response to a stimulus. And experiments on the alligator, where the phenomena can be witnessed on a much larger scale, are equally conclusive in establishing the same doctrine.

"A large alligator (four feet long) being decapitated,* the headless trunk, as on many former occasions, performed numerous actions, indicative of sensation, intelligence, and volition. Resting perfectly quiet, deprived

---

* The division must have been made about the origin of the par vagum, as Dr. Dowler informs us that respiration, though irregular, continued actually 30 minutes by estimation, after removal of the encephalon.

It is a well-known fact in physiology that respiration continues in a decapitated animal only when the origin of the par vagum has been respected.

"Si ce point est detruit, tous les mouvements respiratoires sont abolis sur-le-champ; s'il reste uni à l'encephale, ce sont le mouvements respiratoires de la tête qui survivent; s'il reste uni à la moelle épinière, ce sont, au contraire, les mouvements respiratoires du trônc qui survivent.

"Et enfin, pour ce qui est des reptiles en particulier, ces animaux ne continuent de *respirer*, et de vivre après la decapitation, qu'autant que la section passé sur le crâne et non derrière le crâne, c'est a dire, en autre termes, et en termes plus précis, qu'autant que l'origine de la huitième paire a de respectée, et qu'ayant ete respectée, elle est, de plus, restée une à la moelle épinière."—*Recherches Expérimentales sur les Proprietes et les Fonctions du Système Nerveux. Par P. Flourens*, pp. 424–425.

of all the special senses, it possessed only the general sense of touch, and responded in an accurate manner to all tactile impressions, even the simplest. No extreme agent, neither pricking nor fire, was required to elicit definite and defined movements. The slightest touch with the finger seemed to be perceived by the whole trunk, the tail, and limbs, as perceived by their movements. The animal seemed to be aware of the nature of the touching body, which, if producing little irritation, was borne without any violent efforts to escape from it. But fire, punctures, &c., called into agonized action the body, limbs, and tail. The body curved in a manner so as to recede from the offending agent, and the limbs were directed so as to remove it. From its actions, far more impressive than words, it was evident that it judged accurately, as to the degree, duration, and place of painful or painless impressions."—*Dr. Dowler on Nervous Action.*

We perceive from these experiments that an animal is capable of perception and the manifestation of volition, after being deprived of its cerebrum; and that it is not necessary that the impressions received by the nerves should reach the cerebrum that sensations may be produced.

The animal leaps on being touched, or raises its foot and scratches the part of its integuments that is irritated; or, if an alligator, directs its limbs to remove the cause of irritation, and judges most accurately concerning the stimulus to which it is subjected. And to assert that these are not senso-volitional movements, is to give a new definition to the term. There is in these acts—1, an evidence that the animal has perceived the impressions made on the extremities of the sensory nerves; and 2, a proof that it exerts distinct control over the muscles of voluntary motion, in regulating and directing them to the attainment of a specific end. Because if the animal did not feel, and had no power of volition, why should one limb be raised up, in this manner, in preference to every other, to the part that we touch with the needle? Or why should the hind foot be moved forward to the identical spot in the dorsal region that is irritated? Movements perfectly similar to what we observe in other animals, when a particular part of their body is irritated.

It is no doubt true that the animal has lost the power of spontaneous motion, as it does not move till irritated. And it does not retain the recollection of impressions received by the nerves, as it does not learn to avoid the obstacles to its progress—functions which it is admitted belong to the cerebrum. But, in the absence of these powers, the animal evinces such design, and adaptation of means to ends in resisting every irritation applied to its body, that we must conclude it is capable of distinct perceptive movements.

From these experiments, we deduce the following doctrines:—

I. That the spinal cord enables an animal to feel, and manifest its perceptions by performing definite and combined movements in response to a stimulus.

II. That the cerebrum is superimposed on the spinal cord to act on the possession of these powers, and as the seat of memory and the intellectual faculties.

III. That the associated movements of deglutition, respiration, sneezing, coughing, contraction of the iris, &c., depend on the principle of reflex action possessed by the spinal cord.

It will be observed, from the preceding experiments, that our researches have been confined to the functions of the entire spinal cord, and not of a separate section; as our object has been principally to ascertain if the movements performed by an animal after removal of the cerebrum be illustrative or not of the theory of an excito-motory system. And we consider that the phenomena we have described satisfactorily establish the doctrine that cold-blooded animals, after ablation of the cerebrum, are capable of sensation, and of performing distinct perceptive movements, on the application of a stimulus. And this is the proposition for which we contend.

But another important subject presents itself for consideration: what is the particular seat of this power? Does it belong to the medulla oblongata and upper portion of the spinal cord?

From the experiments we have performed on this subject, in dividing the spinal cord immediately behind the cranium, and removing every portion of the encephalon anterior to this, destroying at once the function of respiration, we maintain that the movements performed by an animal in this condition, on being irritated, afford distinct evidence of perceptive power.*

A very important effect is produced by this division of the spinal cord, as the animal has now lost the power of progressive motion, and is unable to move about and change its place on being irritated. In the former experiments, we have seen that on removal of the cerebrum, the animal

* We may state that in cold-blooded animals, the medulla oblongata is situated at the posterior part of the cranium, and even out of the cranium. In frogs the par vagum is the last pair of cerebral nerves; and the first pair of spinal nerves, and a branch from the second pair, perform functions similar to the par vagum, being distributed to the tongue and the larynx. So that this portion of the spinal cord appears analogous to the medulla oblongata in the higher order of animals. This is still more the case in salamanders.

"Dans la grenouille, l'origine de la huitième paire est place tout-à-fait à la partie posterieure du crâne, a une ligne à peu près en arrière du cervelet.

"La seulė difference qui se trouve, sous ce rapport, entre les animaux a sang chaud, et les animaux a sang froid, est que, dans les premiers, *ce point* est place assez avant dans l'interieur du crâne, et qu'il est, au contraire place tout-à-fait à la partie posterieure du crâne, il presque hors du crâne dans les seconds."—*Flourens.*

loses the power of spontaneous motion. But on division of the cord immediately behind the cranium, the animal is no longer able to perform progressive motion,* but remains in the situation in which it is placed, with its hind legs drawn up, the posture that frogs assume when they rest;† but continues to recognize every irritation applied to its body. Its movements are of a special and determinate character, and identically the same as those which we have described in the preceding section, but performed with less power and energy; while it is also acknowledged that reflex action may be distinctly excited.

These phenomena, however, have been more frequently viewed as a proof of the existence of sensation than of perception. Accordingly, this is the view which Schrœder Van der Kolk takes of the subject, when he considers that the medulla oblongata is the seat of sensation. But, as already stated, we do not believe that we have any proper criterion for determining the existence of sensation in animals subjected to experiment, except the manifestation of perception. For the movements of an animal are the signs by which its feelings and sensations are manifested to us, and we can only properly determine that there is sensation, when we have distinct evidence of perceptive power. And this, we conceive, is afforded us by the following experiments.

*Experiment* 1.—I divided the medulla oblongata of a frog immediately behind the cranium, and reviewed every portion of the encephalon anterior to this. Respiration ceased. Having allowed the frog to remain quiescent for a short time, till the effects of the operation had subsided, I irritated the extremities when the movements appeared most characteristic of design and adaptation. But the irritation caused the frog to perform progressive motion, as in the former experiments, so that a particular effect was produced by this division of the cord, viz. that the animal had lost the power of locomotion, and was unable to move from the place where it was sitting, as was the case with frogs when merely deprived of the cerebrum. I irritated, with the point of a needle, a toe of its left fore foot, and it quickly

---

* I may state that these experiments always succeed best early in spring, when the animal has just left its winter quarters, as its physiological condition of a cold-blooded animal is then particularly developed.

† Je coupée la moelle allongée en tracers, sur une grenouille. J'avais porté l'instrument sur la partie posterieure du crâne.

Tous les mouvements inspiratoires furent abolis sur-le-champ.

L'animal, qui ne respirait plus, continua de vivre pendant plusieurs heures.

Si on le mettait sur le dos, il y restait. Il ne bougeait plus, ou presque plus, de lui-même; mais si ou l'irritait, il s'agitait; si l'on piquait ses pattes, il les reterait, etc.—*Flourens, Recherches Expérimentales, sur les Proprietes et les Fonctions du Systême Nerveux*, pp. 416–417.

withdrew it and placed it below its thorax. I compressed with the point of the forceps a toe of its right fore foot, and it raised up its shoulder and endeavored to withdraw its foot, but being retained, it placed the toes of its right hind foot over the point of the instrument, and threw its body in the opposite direction. After a short interval, I irritated the integuments of its left dorsal region, and it scratched the part with its left hind foot.

*Experiment* 2.—I divided the medulla oblongata of a frog immediately behind the skull, and removed every portion of the encephalon anterior to this. The section was made over the origin of the first pair of spinal nerves which are distributed to the larynx and the tongue. Respiration instantly ceased. I allowed the frog to remain at rest for a short time, till the effects of the operation had subsided, and then observed the phenomena. I compressed a toe of its hind foot with the forceps, and it withdrew its foot and placed it below its abdomen. I irritated the integuments of its right dorsal region, and it raised up its right hind leg and scratched the part with its foot, and then raised itself up and made an effort to move forward. I seized the integuments of the right side of its abdomen with the forceps, and it raised itself up as if to withdraw the part, but being retained, it placed both the fore and hind leg of that side over the part that was irritated. On touching its cloaca with the point of a needle, it drew up both its hind legs over the part that was irritated. I irritated slightly the integuments of its left cervical region, and it raised up its left hind leg and scratched the part with its foot.

*Experiment* 3.—I divided the spinal cord of a frog, at the origin of the second pair of spinal nerves, which sends a branch to the larynx, and removed the encephalon, and portion of the cord anterior to this, as formerly. After a short time I applied irritation, and it was observed that only a slight movement could be produced in the anterior extremities, on account of the injury inflicted on the second pair of spinal nerves, which in the frog go to the fore legs. But on touching its hind foot with a needle, it immediately withdrew it from the source of irritation. On irritating the groin, it drew the thigh into closer contact with its body. On seizing the integuments of the side of its abdomen with the forceps, it moved forward the hind leg of that side and scratched the part with its foot. On irritating the integuments of its abdomen on the opposite side of its body, it moved forward its hind foot forcibly to the part, drawing its body in the opposite direction. I then seized the upper part of the dorsal region on the right side with the forceps, and it actually raised up its hind leg and scratched the part with its foot. I continued the irritation, and it repeated the movement. I next touched with the point of a needle the left dorsal region, and it raised up its left hind leg and passed its toes over the part.

I have repeatedly observed the same phenomena on a similar division of the spinal cord; the animal affording evidence of the recognition of the stimulus, by raising up its leg and scratching with its foot the part that was irritated. But an interval frequently required to elapse before the irritation was renewed, as the animal was weak, and its nervous energy soon exhausted. But on a lower division of the cord the phenomena became much less distinctly developed, and in the inferior portion of the cord we could seldom excite any but a few spasmodic movements of the limbs on applying irritation to the extremeties.

These experiments, we conceive, warrant us to conclude that cold-blooded animals, after decapitation are capable of recognizing a stimulus, and performing distinct perceptive movements, and that this power is seated in the medulla oblongata, or upper portion of the spinal cord. When an animal, after losing the power of locomotion, lifts its foot on being touched with a needle, and places it below its thorax or abdomen, or raises it up and repeatedly scratches the part of the integuments that is irritated, we must admit that it feels pain, and perceives the source of irritation. In short, that it manifests cognizance of the stimulus, by the definite and combined movements that it performs.

But this view of the subject has been objected to by some physiologists on the ground that as we have distinct evidence of design and adaptation of means to ends in many of the reflex movements of the body, such as contraction of the iris, the associated movements of sneezing, coughing, defecation, &c., so the movements of design manifested by a decapitated animal when it lifts its foot and scratches off any cause of irritation from its back, &c., may be of the same character. But there is a great and manifest distinction between the two cases. In the contraction of the iris, and the associated movements of sneezing, coughing, &c., we have an adaptation in the structure of the parts to the particular function which they perform, or an evidence of design in the Author of nature endowing these parts with the property of contracting on the application of a particular stimulus, and to the attainment of a specific end, just as we observe in the contraction of the heart, &c. But these movements afford no evidence of the adaptation being the design of the animal, as they are merely a uniform sequence or contraction of the parts from a particular irritation; every successive movement being a repetition of the former, and performed without change or variation, these cannot be considered a proof of perceptive power. But when an animal lifts its foot to scratch off any cause of irritation from its back, it does not result from any particular structure of the parts to that particular end, as that is only one of a multitude of movements which it might have performed; thence the evidence which it affords is that of being a perceptive act, or of design on the part of the animal. And this has universally been considered the data by which we determine the character of a perceptive movement in an unmutilated animal. When we, therefore, see a decapitated frog lift its foot in a similar manner, and scratch the part that is irritated, are we not bound to conclude that we have here as distinct evidence of perceptive power as in the former case, and that the movements are identically the same? For if this be not a perceptive movement, it undoubtedly bears all the characteristics of those that have otherwise been considered as such. And then we must admit that in a decapitated animal a definite end is accomplished by indefinite means, which certainly appears to be very remarkable.

There are other remarkable phenomena observed in experiments on the salamander, to which we must refer in corroboration of the views we maintain, and as decidedly opposed to the theory of an excite-motory system. The abettors of that system consider that after the spinal cord is cut off from all influence and communication with the brain, it is only reflex movements that can be produced, and that this is also the case with distinct sections of the cord. When, for example, the spinal cord of a higher order of vertebrated animal is divided below the brachial plexus, the animal is immediately seized with paralysis in its lower extremities; and the reflex movements excited in the part by irritation of the integuments, are of a general and indefinite character, and consist of alternate contraction and extension of the limbs. But when the spinal cord of a salamander is divided below the brachial plexus, the animal does not suffer paralysis in its lower extremities, but continues to move them as before, exerting distinct power over them, as in the act of locomotion, lifting up one foot and then the other, in accordance with the movements of its fore legs, as it crawls slowly along the surface of the table.*

If the excito-motory theory be based on fact—if it be a general law in vertebrated animals that the spinal cord is capable only of reflex action, how does it happen that the animal is able to perform the movements of locomotion after division of the spinal cord?

I took a salamander (*Lacuta aquatica*, Linn.) and divided the vertebræ and spinal cord immediately below the brachial plexus, which, in that animal, as in the frog, is the second pair of spinal nerves, and sends a branch

* Flourens, who holds the theory of the excitability of the spinal cord, admits that the salamander retains the power of locomotion in its hind feet, after division of the spinal cord below the fore arms, and endeavors to account for phenomena so much at variance with his views on the principle of reproduction of parts. But how could that principle confer this positive power upon the animal, if it were not seated in the spinal cord at the period that the section was made?—*Flourens, p.* 419.

to the larynx;* so that the animal retained complete power over the movements of its anterior extremities; and after allowing the animal to remain quiescent for a short time, till the effects of the operation had subsided, observed the phenomena.

The animal raised itself upon its fore legs and began to move forward, but did not drag its hind feet like an animal that had suffered paralysis, but supported its body on them as on its fore legs, and exerted them distinctly in the act of locomotion. I could observe no difference between these movements and those which it performed before the division of the cord, except that it now walked with less power and energy. I allowed the animal to remain at rest for a short time, and then slightly touched with the point of a needle the integuments of the right dorsal region, and it raised up its right hind foot and passed its toes across the part. On irritating the integuments of the left side of its abdomen, it raised up its left hind foot again and again to the part. After a short interval, I touched with the point of a needle the upper portion of the left dorsal region immediately below the division of the cord, and it raised up its left hind foot and passed its toes distinctly over the part. I continued the irritation, and the animal repeated the movement, raising up its left hind foot and passing its toes over the part.

*Experiment* 4.—I took a salamander (*Lacuta aquatica,* Linn.) and divided the vertebræ and spinal cord immediately below the brachial plexus, allowing the animal to retain perfect power over its anterior extremities. I then removed a small portion of the vertebræ from the lower division of the cord, so that the divided ends remained distinct and separate, and observed the phenomena.

After the animal had remained at rest for a short period, it raised itself upon its fore legs and commenced to walk, and exerted its hind legs most distinctly in moving along the table. It raised up one hind leg and threw it forward, while it retained the other in its position, in correspondence with the movements of its fore legs, as it performed locomotion. But it walked more slowly and with less vigor than before the division of the cord, and during this period the divided ends of the cord remained distinct and separate. I touched with the point of a needle a toe of its right hind foot, and it withdrew it and placed it along the side of its abdomen. I irritated the integuments of its right dorsal region, and it raised up its right hind leg and passed its toes over the part. I touched slightly with the point of a needle the integuments of the left dorsal region, a little be-

* In salamanders, as in frogs, the upper portion of the spinal cord appears analogous to the medulla oblongata in the higher order of animals, as the first pair of spinal nerves and a branch from the second pair proceed to the larynx and the tongue.

low the division of the cord, and the animal raised up its left hind foot quickly to the part. I continued the irritation, and it passed its toes distinctly over the part; but it did not move its posterior extremities till a part of its integuments was irritated, and then it gave the most unequivocal evidence of recognizing the impression.

I have performed the same experiments on many other salamanders with similar results,* and I have, after division of the spinal cord below the origin of the brachial plexus, removed the upper portion of the cord as far as the occiput, destroying all power of motion and sensation in its anterior extremities; and on touching with the point of a needle the integuments supplied with nerves from below the division of the cord, the animal has raised up its hind foot and passed its toes over the part. I have, after this, removed the encephalon, and on applying irritation to the integuments, as the side of its abdomen or dorsal region, the animal has still continued to recognize the stimulus—has raised up its foot and passed its toes distinctly over the part that was irritated. But the movements were weaker, and an interval required to elapse before they could be renewed.

On a lower division of the cord, this power becomes less manifest, and the reflex action more particularly developed.

We are anxious to urge these facts on the consideration of the profession, as our object is to elicit the truth, and determine the specific functions of the spinal cord, irrespective of any particular views. And we think we are entitled to demand an explanation of these facts from the abettors of an excito-motory system, and to ask on what principle they account for the phenomena. The movements must either be reflex or they must not. If the former, then how do they differ so essentially from all reflex movements that have been recorded, as in none of these cases has the power of locomotion been retained on a separate and similar division of the spinal cord. And if they are not reflex, how do the supporters of the theory maintain their views after the manifestation of such phenomena? We, therefore, consider it an established fact in physiology, that in salamanders, after the division of the spinal cord below the brachial plexus or second pair of spinal nerves, the animal manifests recognition of an impression, and distinct power over the movements of its posterior extremities in locomotion.

If we maintain that the spinal cord is constructed on the same type in vertebrated animals, we must arrive at the general conclusion that there is a power resident in the medulla oblongata, or upper portion of the spinal

---

* In a paper which I published in the Edinburgh Medical and Surgical Journal, No. 167, I have given a full account of similar experiments performed on the salamander, and their results.

cord, which enables an animal to recognize impressions, and perform movements in response to them, and that this power is seated lower in the cord in some classes of cold-blooded animals than in others.

In conclusion, we may state that we have endeavored to establish our views on the principles of inductive science, by clear and distinct experiment, and we must interpret the laws of nature according to the facts observed, and not according to any preconceived opinion respecting the nature of mental phenomena. But these doctrines are in no respect opposed to the unity of mind, a subject much insisted on by some physiologists. For while the spinal cord enables an animal to receive impressions, and perform movements in response to them, it has no power to originate an action in itself—that belongs to the cerebrum. Hence the brain is superimposed on the spinal cord, to act on the possession of that power, and as the seat of memory and the intellectual faculties.

